# Feeling Heard: Experiences of Listening (or Not) at Work

**DOI:** 10.3389/fpsyg.2021.659087

**Published:** 2021-07-26

**Authors:** Tiffany D. Kriz, Avraham N. Kluger, Christopher J. Lyddy

**Affiliations:** ^1^Department of Organizational Behaviour, Human Resources Management, and Management, MacEwan University, Edmonton, AB, Canada; ^2^Department of Organizational Behavior, School of Business Administration, The Hebrew University, Jerusalem, Israel; ^3^Department of Management, School of Business, Providence College, Providence, RI, United States

**Keywords:** listening, perceived listening, communication, qualitative, non-listening, feeling heard

## Abstract

Listening has been identified as a key workplace skill, important for ensuring high-quality communication, building relationships, and motivating employees. However, recent research has increasingly suggested that speaker perceptions of good listening do not necessarily align with researcher or listener conceptions of good listening. While many of the benefits of workplace listening rely on employees feeling heard, little is known about what constitutes this subjective perception. To better understand what leaves employees feeling heard or unheard, we conducted 41 interviews with bank employees, who collectively provided 81 stories about listening interactions they had experienced at work. Whereas, prior research has typically characterized listening as something that is perceived through responsive behaviors within conversation, our findings suggest conversational behaviors alone are often insufficient to distinguish between stories of feeling heard vs. feeling unheard. Instead, our interviewees felt heard or unheard only when listeners met their subjective needs and expectations. Sometimes their needs and expectations could be fulfilled through conversation alone, and other times action was required. Notably, what would be categorized objectively as good listening during an initial conversation could be later counteracted by a failure to follow-through in ways expected by the speaker. In concert, these findings contribute to both theory and practice by clarifying how listening behaviors take on meaning from the speakers' perspective and the circumstances under which action is integral to feeling heard. Moreover, they point toward the various ways listeners can engage to help speakers feel heard in critical conversations.

## Introduction

“*He wasn't listening. And it eventually got to the point where…when he would ask a question, I would just give him what he wanted to hear. So really it wasn't a true feedback from me. … If I can't get anywhere with you, then why even bother?*”—Interviewee Greg

Organizations rely on good communication to foster high-quality relationships and realize performance goals. However, communication breakdowns occur frequently, and can impose significant costs (Maxfield, 2016). The perception of listening may play a role in these communication breakdowns. A tragic example comes from the explosions on the Challenger and Columbia space shuttles, disasters that may have be avoided through better listening. “In both cases, engineers initially presented concerns as well as possible solutions… Management did not listen to what their engineers were telling them” (CAIB, 2003, p. 201). The failed information exchange in these examples were catastrophic, yet perhaps the outcomes would have been different if the engineers continued trying to share their concerns after the initial failed efforts. Unfortunately, records indicate the engineers gave up after their concerns went unheeded—much like Greg in the quote above.

Both scholarly research and practical wisdom now suggests that organizations benefit from high-quality listening. A recent review on the topic suggests that beyond impacts on willingness to speak up about critical issues, poor listening can result in other detrimental outcomes such as turnover, burnout, job dissatisfaction, and low commitment, whereas high-quality listening can strengthen relationships and lead to better outcomes for individuals and organizations (for review, see Pery et al., 2020). Accordingly, there is a strong practical imperative to improve the quality of listening efforts (Itzchakov and Kluger, 2018). Yet as the importance of listening becomes clearer, so too have the gaps in knowledge which could threaten the scholarly and practical treatment of listening in the workplace.

The most appropriate perspective of listening to assess depends on the aims of a particular research study (Bodie et al., 2014; Worthington and Bodie, 2018). Within the realm of workplace listening research, scholars are often focused on the speaker's perception of dyadic listening due to the importance of this perspective in driving key workplace outcomes (see review by Pery et al., 2020). For example, Brownell (1990, p. 403) argued that “the **perception** [emphasis in original] of effective listening is vital.… When employees say their manager ‘doesn't listen’ it is essential to know what this means.” Yet most workplace research, rather than starting from the perspective of employees' lived experiences on listening, has used either experimental listening manipulations or survey methodology, each typically based on researcher-derived conceptions of good listening. Notably, there are exceptions in which researchers have built conceptions of listening based on actual accounts of dyadic workplace interactions (e.g., Lewis and Reinsch, 1988; Lipetz et al., 2020). Yet while these exceptions create greater insight into the subjective perception of workplace listening, the decontextualized lists of behaviors they offer are not suited to provide insight into how the items take on meaning for speakers; rather, qualitative, inductive studies are better suited to this purpose (Maxwell, 1996). Accordingly, it is currently unclear how well researcher conceptions of listening map onto the subjective experience of being listened to in the workplace.

Indeed, a lack of insight into speakers' subjective experience of workplace listening appears to be problematic because listeners' self-perceptions of listening, as well as objective ratings of listening, can each differ substantially from speakers' perceptions (Hunt and Cusella, 1983; Brownell, 1990; Bodie et al., 2014). This suggests that the experience of being listened to is not readily observed by external parties, even when they are attentive observers or even participants in a conversation. Accordingly, clarifying this experience could inform a richer conception of subjectively perceived workplace listening, offering insights for theory and measurement of perceived listening from the speaker's perspective. From a practical perspective, understanding subjective perceptions of listening interactions should pave the way for more effective interventions (Brockner and Sherman, 2019).

Some workplace interactions could be more important than others in shaping outcomes and perceptions of another's listening. Relational perceptions and dynamics can become permanently altered based on certain defining moments (Ballinger and Rockmann, 2010), such as how a supervisor reacts to a pregnancy disclosure (Little et al., 2017). Likewise, the way in which listeners are perceived to respond at key times can shape larger sensemaking around their inclusion in the workplace, having downstream effects on outcomes such as organizational commitment and turnover intentions (Reynolds-Kueny and Shoss, 2020). Accordingly, interactions holding subjective importance to a speaker are likely to hold a more prominent place in driving perceptions of listening and shaping subsequent attitudes and behaviors.

To investigate how individuals subjectively experience important workplace listening interactions, we conducted interviews with bank employees, asking about past interactions in which someone had an important opportunity to listen to them at work. Through our analysis of these interviews, we were able to gain insight into the various paths through which employees came to feel heard or unheard at work, and the circumstances under which certain listener behaviors took on meaning for employees. Before moving on to provide a detailed account of our methods and findings, we first provide a review of the relevant scholarly and practitioner accounts of listening upon which our work builds^1^.

## The Nature and Importance of Listening

Listening is a widely valued skill across academic and non-academic domains. There is no consensus among scholars on the definition of listening, however, scholars agree listening is multi-faceted and tends to encompass “(a) affective processes, such as being motivated to attend to others; (b) behavioral processes, such as responding with verbal and non-verbal feedback; and (c) cognitive processes, such as attending to, understanding, receiving, and interpreting content and relational messages” (Worthington and Bodie, 2018, p. 3). Defining listening more precisely has proven difficult for scholars as the term tends to take on different meanings depending on who is using it, and in which context they are using the term (Worthington and Bodie, 2018).

Early scholarship on listening focused on lecture comprehension in students, framing listening as a way to make use of information conveyed in communication (Nichols, 1948; see also review by Bodie, 2012). Around the same time, Carl Rogers was beginning to write about the importance of listening within therapeutic relationships, pointing toward the role of good listening in enabling clients to achieve transformational personal change (Rogers, 1959). More recently, scholars have pointed toward the implications of listening in a storytelling context for shaping memories of prior events, and as such, shaping one's sense of self (Pasupathi, 2001; Pasupathi and Billitteri, 2015). Each of these areas views listening from a different angle, yet all view it as a key process with an ability to shape the way an individual sees the world. Likewise, researchers across several domains have framed listening as something that happens in interpersonal relationships with strong implications for the development and maintenance of those relationships (e.g., Kluger et al., 2020). Thus, scholarly and practitioner attention toward listening has persisted despite difficulty in pointing toward a single definition or theoretical framework emcompassing the nature and importance of listening.

Practitioner attention toward listening tends to emphasize changing behaviors—a fitting focus as listening is perceived behaviorally (Witkin, 1990). Among the behaviors that were suggested by Carl Rogers was active listening (Rogers and Farson, 2015), popularized in leadership training by one of his students (Gordon, 1977). Active listening is now considered as a set of behaviors that in concert create good listening. These behaviors include quieting the mind before listening, or practicing mindfulness (Friedman, 2005), asking open-ended questions (Nemec et al., 2017; Van Quaquebeke and Felps, 2018), paraphrasing and reflecting feelings (Nemec et al., 2017), and validating (Linehan, 1997).

Importantly, a behavior emphasis in training does not necessarily translate into any positive consequences. For example, couples can be trained to engage in active listening but marital satisfaction may not be affected (Garland, 1981). Similarly, Rautalinko and Lisper (2004) found that insurance employees trained in listening displayed more listening behaviors post-training, but were not evaluated any differently than those who were untrained. These results seem to suggest that it is not just the behaviors that matter to effective listening, but how those behaviors are perceived. Interestingly, as part of a multitrait-multimethod analysis, Bodie et al. (2014) recorded a listener and speaker having a 5-min conversation, then asked the listener, speaker, and a trained coder each to rate the listener's behavior, finding only a moderate correlation (*r* = 0.30) between speaker and coder ratings of the same interaction, and a negative correlation (*r* = −0.06) between listener self-assessment and speaker ratings. Given that it is typically the speaker's reaction to the listener that drives important outcomes (Pery et al., 2020), such findings point toward the need to better understand what is encompassed within the speaker's subjective perception of a given listening interaction.

Some form of conversational responsiveness is likely to be important to perceived listening. Bavelas and colleagues observed how as listeners and speakers respond to each other's conversational signals to accomplish the co-construction of storytelling (Bavelas et al., 2000), finding that responsiveness came in the form of both verbal and non-verbal responses, and could be specific to the content shared or generic utterances (e.g., “uh-huh”) signaling that the listener is paying attention and providing encouragement to the speaker to continue. Many of these responses, especially those that are more tailored to the speaker's stories, also serve the function of demonstrating understanding (Bavelas and Gerwing, 2011), a key indicator of good listening (e.g., Lipetz et al., 2020). Such responses can also include questions (Bavelas et al., 2000), which can promote further understanding while also signaling interest and attention (Van Quaquebeke and Felps, 2018). As this work suggests, listener responses within conversation are an important part of what listeners do and how they are perceived.

Although listening is typically viewed as a process taking place within a given conversation, findings from two studies have suggested that listening in the workplace context could potentially encompass responsive behaviors outside conversational boundaries. In the first, researchers content analyzed accounts of effective and ineffective listening, identifying a list of 38 categories of listener behavior observed or reported by participants, three of which involved behavioral responses demonstrated by the listener including both immediate and delayed actions, including “did (or did not) follow my directions or suggestions” “Did not (or did) ignore my message or not react to it,” and “Did (or did not) try to get changes made or results I requested” (Lewis and Reinsch, 1988, p. 56). Second, Kocoglu et al. (2019) theorized and found empirical support for the idea that although action may not be central to listening perceptions in a dyadic context, it is critical to the demonstration of listening in a team context. In summary, although action is not typically defined as being within the boundary of listening (or perceived listening), these studies raise questions about a potential role for action in workplace perceptions of listening.

### Current Study

Our research aimed to understand the essence of feeling heard at work and what factors lead to this experience. More specifically, we sought to understand (1) how do individuals explain their journey to arriving at feeling heard or unheard in prior listening interactions, and (2) what factors differentiate feeling heard from feeling unheard in prior listening interactions?

### Setting and Approach

This study was conducted on-site at a Midwestern USA bank. Over the past decade, the banking industry has encountered several changes in its environment, which have required adaptation, such as increased regulation and increased online banking. The adaptations included creating new roles and procedures, increasing the importance of staying in touch with the needs of those on the front lines. This study's particular bank has been recognized for its developmental approach to employees, thus promising a fertile ground from which stories of good listening could emerge.

We conducted semi-structured, in-depth critical incident interviews with 42 bank employees. Approximately two-thirds of the interviews were conducted at the headquarters location and the remainder at branch locations in a separate region. We used in-depth interviews because the perspective of the employee was of paramount importance to answering the research questions (Ritchie and Lewis, 2003), and invited a storytelling approach to capture detailed information about significant events that occurred in the past (Coffey and Atkinson, 1996; Boyatzis, 1998).

## Methods

### Sample

We used purposive sampling to identify potential participants (Maxwell, 1996; Miles et al., 2014). With the assistance of the bank's human relations (HR) department, we identified a pool of potential interviewees from several different positions, functions, and locations (Guba and Lincoln, 1994). We asked that HR select for interviews only those individuals they considered to be mid- to high-performing, because they are likely to be viewed more positively than their lower-performing counterparts and therefore more likely able to tell stories about managerial listening (Ashford et al., 2009). We interviewed only employees who worked in relatively interdependent roles because they were most likely to provide rich insights on the experience of listening at work.

Out of 50 targeted employees, 42 consented to participate (24 female), while the other eight were either unreachable by the researcher or could not participate due to scheduling difficulties. Data from one interview could not be used due to a lost audio recording. Nearly half of the employees interviewed represented retail-banking centers, which served individuals and small businesses. Nine individuals worked in the investment-advisory arm, five individuals worked in wholesale banking (serving mostly larger businesses), and eight worked in other areas (e.g., marketing, HR). All participants reported that they worked for the bank full-time, and that English was their first language. Table 1 displays aggregate demographic data.

**Table 1:** Interviewee demographic data.

**Variable**	**Mean (SD) or count (%)**
Interview length (minutes)	43.9 (11.7)
Gender	
Female	24 (59%)
Male	17 (41%)
Age	41.1 (10.1)
Unknown	1
Tenure in organization (months)	113.0 (87.4)
Tenure in position (months)	57.9 (51.0)
Education	
High school/GED	1 (2%)
Some college	6 (15%)
2-year college degree	3 (7%)
4-year college degree	23 (56%)
Master's degree	8 (20%)

*N = 41*.

### Interviews

Before beginning each interview, the first author gave interviewees a broad overview of the research and provided time to review, ask questions about, and sign the informed consent form. Interviewees were also asked separately for permission to audio-record the interview; all but one participant agreed. Participants were briefed on measures to maintain their anonymity. Specifically, only the first author and a 3rd-party transcriptionist service would be allowed access to their raw, identifiable interview data, identification codes would replace identifiable the data on notes and demographic form, and identifiable details (e.g., names) would be removed or altered prior to publication or sharing any details with their employing organization. After providing consent, interviewees filled out a brief demographic questionnaire asking about birth year, primary language (English or other), work status (full time, part time >20 hours per week, or part time working <20 hours per week), tenure within the organization and position, and level of education.

We developed and used an interview protocol asking about critical incidents involving workplace listening interactions (Flanagan, 1954). Our protocol (see Supplementary File) asked about both listening and non-listening events to enable comparisons between the two types of stories (Boyatzis, 1998). The main interview questions were “Tell me about a time when someone at work had an important opportunity to listen to you, and he/she took that opportunity” and “Tell me about a time when someone at work had an important opportunity to listen to you, but he/she failed to take advantage of that opportunity.” General probes were prepared to follow-up with interviewees on points needing further elaboration. These probes served to ensure that stories provided similar types of data across interviews, and more specific follow-up questions were used as needed to better understand the events and personal meaning attached to the specific details conveyed in a given story (Kvale, 1996). Questions continued as needed until the point of data saturation, achieving as full an understanding of the storyteller's perspective as possible (Ritchie and Lewis, 2003). The 41 participants collectively provided 81 stories, of which they designated 47 stories of feeling heard and 34 stories of feeling unheard. Interviews ranged from 21 to 72 min in length in total (average time was 44 min).

## Analytical Process

The interviewing, coding, and analysis were conducted iteratively, during and after the interview period (Miles et al., 2014). Throughout the coding and analytic process, the interviewer kept notes and analytic memos, returning to interview observation notes for additional context as needed (Miles et al., 2014). Our research questions required both tactics of both categorizing (e.g., coding discrete pieces of text) and contextualizing (e.g., examining contextualized data within each case to determine causal flow), each of which requires a different analytic approach (Maxwell, 1996; Miles et al., 2014). Accordingly, our analysis started out with open coding of each story (a categorizing tactic) followed later by analyzing narrative structure of each story to determine how the storyteller arrived at feeling heard or unheard (a contextualizing tactic). NVivo 10 was used to store transcripts, separately tag stories within each transcript for later search and retrieval, and complete line-by-line coding. Microsoft Excel was used to create story summaries that could be examined alongside other information at the story level for contextualizing purposes. Moving back and forth between categorizing and contextualizing, each tactic granted an increasing amount of clarity to the other. Eventually we were able to link both strategies through the creation of a composite sequence analysis, which is a way to identify commonalities in multiple participant journeys or paths (Maxwell, 1996; Miles et al., 2014)—in our case, paths toward feeling heard or unheard.

### Story Coding

The researcher first read each transcript and, based on interviewees' explicit classifications, coded each distinct story within interviews as either a listening story or a non-listening story. Stories that came in response to the question about someone who *took* an opportunity to listen were thus categorized as *listening stories*, whereas stories that came in response to the question about someone who *did not take* an opportunity to listen were categorized as *non-listening stories*. In some cases, interviewees told stories of non-listening but added further detail about how, in the end, they tried again and experienced a better result. These cases were categorized as listening stories if they were told in response to the listening story question or non-listening if they were told in response to the question about a failed listening opportunity.

In many cases, interviewees told stories involving multiple listeners. If two listeners acted in drastically different ways, with each garnering roughly equal attention from the storyteller, they were classified as two separate listening stories. In general, most stories involved one or more higher-ranking individuals and a majority of stories referenced interactions that took place through multiple interactions over time.

Stories were coded for content using open coding. This initial coding effort was applied to each story on a line-by-line basis while staying close to the original language of the participants. This process resulted in 181 unique codes. Because these codes were developed inductively, the list expanded and evolved with each new story coded and these codes were further developed and refined with continued reading and re-reading the stories. We attached basic descriptive labels to group these codes into categories, such as triggering events, outcome, listener, responding (listener behavior), and context. The descriptive labels attached to codes at this point were tentative labels used for the purpose of finding similar types of codes before creating a new code (to prevent further proliferation) or were primarily used for indexing purposes.

Early on, each story typically contained several different codes relating to listener responsiveness, yet as data collection and analysis continued, it became clear that many of these codes were idiosyncratic in that they did not appear in other stories. Further, analytic memos suggested that many codes did not distinguish between vastly different listening experiences, which was central to our research question. Thus, data condensation was necessary to facilitate comparisons across stories and identify attributes at the story level that might distinguish between varying listening experiences. Toward this end, each story was distilled down to a short summary (Kvale, 1996), which was then entered into a spreadsheet alongside columns reflecting other story properties. These summaries served as reminders of a given story rather than an artifact to be coded. Similarly, we also identified a metaphor for each story, which forced us to start making sense of the data by considering the story as a whole and the most meaningful elements of that story (Miles et al., 2014).

### Differentiating Listening From Non-listening

A matrix spreadsheet was created to compare the stories' similarities and differences and identify patterns and relationships (Miles et al., 2014). This step was intended to identify codes that reliably differentiated listening stories from non-listening stories (Boyatzis, 1998). New descriptive codes were created at the story level, such as duration of the listening exchange (i.e., a single conversation or a series of interactions over time relating to the same initial interaction) and topic initiator (listener or speaker). We compared stories in various ways to identify patterns and differentiators (Miles et al., 2014). Some of the most frequent codes from the line-by-line coding (e.g., listener took action) were first examined as potential differentiators between stories, yet most did not reliably differentiate between stories (e.g., a listener taking action was often observed even in non-listening stories). We tried other ways of grouping the stories (e.g., by sharing topic, conversation initiator—speaker vs. listener), yet again these efforts were futile in terms of reliably differentiating between stories. Many codes were abandoned during this process, including sharing strategy, context, conversation initiator (speaker or listener), and sharing location. Ultimately, the codes that best differentiated between listening and non-listening stories were shutting down behaviors and listener openness. Shutting down was coded if the listener forcefully ended the conversation (e.g., hearing but overruling the speaker without sufficient consideration). Openness was coded if the listener was described as being open to what the employee had to say, or if openness could be implied from the listener taking relevant action based on something the employee said.

Although openness and shutting down behaviors performed better than other categorizations in distinguishing between listening and non-listening stories, a few stories did not fall neatly into one of these categories. For example, Nancy^2^ told a listening story where the listener initially showed defensive shutting down but opened up over time as the speaker re-engaged. On the opposite end, many of the non-listening stories lacked reference to shutting down behaviors. A closer look at these stories indicated that the listener seemed to display some openness upfront but was retrospectively classified by the interviewee as non-listening due to a lack of expected follow-up action. At this stage, we shifted focus away from analyzing all of the listener behaviors within a given story and toward identifying which listener behaviors were most central to the final assessment of listening.

### Classification of Specific Listener Behaviors and the Identification of Story Paths

We employed narrative analysis to identify the primary behavior tied to the final assessment of feeling heard or not. Specifically, the narrative structure was examined to identify the turning point or pinnacle at which the speaker felt fully heard (Parcell and Baker, 2018). This process started by reviewing the story metaphors and summaries for initial clues, which were then thoroughly checked against the full story transcript. This process helped to further clarify the listening behaviors within the context of their meaning in the overall story narrative, which ultimately helped us further refine the labels of those behaviors to better reflect the stories in which they were embedded (Miles et al., 2014).

To better understand the meaning that listener behaviors held for interviewees and how those impacted their experience of feeling heard or unheard, it was necessary to pay attention to latent needs or expectations reflected in interviewees' stories. Through examining listening experiences at the story level it became clearer that the way the interviewee told the story often revealed themes around the speaker's needs and expectations, which shaped their views of listener responsiveness. For example, one interviewee was upset about the rejection of her request (an easily implementable idea with good payoff for the bank) because she saw it as a strategy to help the bank more than it would help her personally. Accordingly, this revealed that she expected her request to be heard and acted upon, and that she had a need to have her ideas treated with the respect and consideration she felt they deserved. Similarly, all non-listening stories involved a violation of listening expectations, whereas listening stories, in contrast, involved an attempt to meet the speaker's need that met—and possibly exceeded—the speaker's expectations. These expectations were often not made explicit by the speakers nor were they necessarily present from the beginning of the listening interaction described. Instead, expectations often emerged as the interaction unfolded; for example, an employee might drop any expectations of action upon learning in conversation that the listener had no power to act on their input.

**Figure 1:**
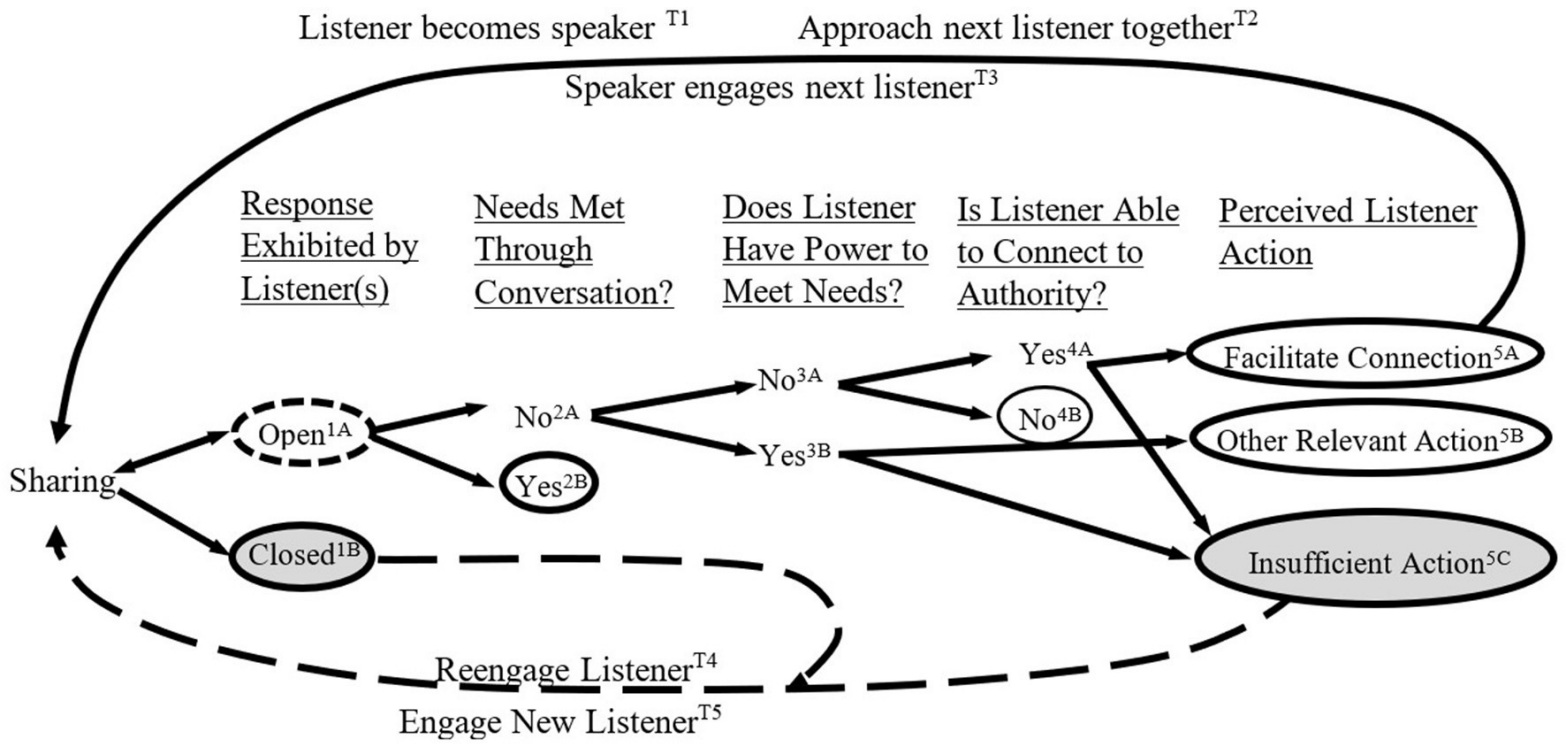
How employees see and judge listening as listening opportunities unfold. Path ending in 1B represents “rejection;” path ending in 2B represents “conversational growth;” paths ending in 4B and 5A represent “tentative listening;” path ending in 5B represents “feeling heard through action” and path ending in 5C represents “disappointment”.

By identifying the central listening behavior tied to the overall experience of listening, we were able to better see how those listening behaviors differentiated between stories, clarifying the various ways of arriving at feeling heard or unheard. Specifically, focusing in on the key listener behavior in each story and examining these behaviors across different stories revealed broader themes encompassing those behaviors (e.g., various forms of listener action). Likewise, we were able to move to the next level of abstraction by seeing how these second-order themes fell into natural grouping (e.g., stories in which the conversation itself was enough to meet the threshold of feeling heard from those in which action was identified as a critical component to the assessment of listening). This process was facilitated through the use of composite sequence analysis (Miles et al., 2014), which involved simultaneous consideration of the contextually-rich information provided within each story and similarities and differences across stories. This process enabled us to identify the various paths through which interviewees, as a collective, could end up feeling heard or unheard (see Figure 1).

Through analyzing the various paths through which listeners came to feel heard or unheard, it became apparent that some listening stories hinged on *building* together through conversation—building an idea, building capabilities through coaching, or building compassionate connections by being with the speaker unexpectedly—either through engaging in conversation around a difficult topic or showing unexpected interest. In contrast, other listening stories hinged on taking relevant action. Accordingly, we classified a story as meeting needs/expectations through conversational *building* if (1) the storyteller classified it as an example of listening, and (2) the storyteller emphasized the conversational discourse as the meaningful element of listening, rather than emphasizing any actions that flowed from that discourse. If the storyteller emphasized follow-up action as necessary to the experience of being listened to, the story was classified as meeting needs through *acting*. Non-listening stories could either be classified as hinging on a *closed* listener response if they were tied to various forms of shutting-down behavior resulting in feeling unheard, or as *insufficient action* if they were tied to unmet expectations around follow-up action. There were two outliers to the emerging types of listening experiences. Specifically, two interviewees told stories in response to the listening prompt in which they expected action from the listener but had not yet seen that action take place at the time of the interview. While this aberration was only seen in two stories, we include these stories in our findings under the category of “tentative listening” in order to provide a full accounting of the various types of listening experiences described by our participants.

As these categories were developed, we constantly compared against the listener behaviors, which helped us to further refine labels and descriptions for those behaviors. Further, we recruited a second coder, external to the research team, to code a random sample of 16 stories, or ~20% of the data. Agreement between the two coders was moderate (Cohen's kappa = 0.58). In order to resolve disagreements, we slightly modified the descriptions of some codes and combined two sets of first-order themes which could not be reliably differentiated. After going through this process we were able to reach full agreement, and the first coder then went back through the full set of stories to recode other stories as needed. In total, 16 categories of listener behaviors were retained, which were subsumed into 9 second-order categories and four aggregate dimensions. Table 2 displays the data structure (Gioia et al., 2012) and representative quotes.

**Table 2:** Data structure including first-order concepts, second-order themes, and aggregate dimensions.

**Aggregate dimensions and second-order themes**	**First-order concepts and illustrative data**
*Closed listener response*	
Shutting down	*Making self-unavailable for discussion* She always tries to avoid conflict [and] she sat in a different [USA] state-so she was able not to have to be involved. (Clarissa) He just wasn't really around (Danielle)
	*Ignoring* I feel like it's heard, but not really. Nothing's going to ever happen. But somebody else might present that information in a very similar fashion in a very similar way, but it may be discussed more. (Dennis) I think in that situation, they just kind of wanted to brush it off because it would have involved an interaction with a high-level person. (Melanie)
	*Hearing but overruling the speaker's perspective* It was more that my pushback was overruled I guess is what I would say. (Jacob) I think that he felt like, hey, I've been in this business a lot longer than you have. I think I know what's best here. I think this is the way we do it. You know? Kind of discounting my experience and my viewpoint. (Tom)
*Building*	
Expanding	*Helping the speaker to build an idea* So then I contacted Yolanda, and we brainstormed and figured out, alright, what do we need to do from here? (Linda) They were very open in listening and working with me in pitching on it…[sharing] different options, different scenarios, different past experiences, things like that. (Jody)
	*Coaching* The conversations are always very much centered around me and what I said… So the conversations over time went from learning the business and learning what it takes to be a private banker to okay now you're doing them, what else do we need to do to make sure whenever we do flip the switch that you hit the ground running. (Jacob) They kind of asked me, “Well, how do you think that went? What do you think was the outcome? What was good? What was not good? I mean, it's just – it's good for me [to discuss observations with seniors] because it's a good experience - because that's what I want to do. (Heidi)
Being with	*Willingness to engage with the speaker around a difficult topic* At the time, I felt, you know, completely on Lone Island. But it was nice for her to be there when I was having a breakdown about it. (Melanie) He took a customer complaint and [instead of] saying, “Why is this happening? What did you do wrong?” he actually asked me to explain to him what was going on, and listened to me. (Jason)
	*Showing interest when it's not expected* It stands out because she's very high level. I feel like when you are at the bottom of the totem pole, then you can get your ideas up to the top, it's really nice. And then maybe some change can happen from it. (John) And [local leader] came out, and she assisted us with our team engagement, and she actually listened to what the employees communicated to her. (Jennifer)
*Acting*	
Using feedback	*Using feedback to make a personal change* She listened to what we had to say, and she made a lot of changes of her own, which made a lot of changes just in general within our entire region. (Nancy) I said if you let me work with you, I said I'm going to get you to where you want to be. And she says okay. And she did everything I told her to do, every single thing. (Andy)
Facilitating good work	*Providing a requested tool* The minute he knew it [malfunctioning copier] was really, really making my job difficult, we had [a new one] within a month. (Felicia) I first walked in and subsequently begin to ask questions, “When are we going to get remodeled, when are we going to get upgraded, when is…” and was relentless. I just got off a conference call yesterday that we'll be breaking ground in about sometime third quarter. (Lauren)
	*Using input to make a change that benefits the organization or other individuals* [My boss] wanted to…lighten up the mood with everyone, and I gave him a suggestion… and he implemented it. (Felicia)
	So in this particular case, I kind of push it and I said, hey guys, why do we need to do this… We could potentially create a negative client impact or client experience… they finally, not necessarily said you're right but said, okay, because of this, we don't have to do that. (Sarah)
	*Work-related solution creating* [My boss] came up with an idea around how to try to keep [a star employee], which involved increasing salary and a chance to manage. It would mean creating a new position. I mean, this is culturally and historically not easy to turn around when you're talking about a significant increase… I would say by two o'clock we had enough buy-in from key stakeholders [to make a counteroffer]. (Tom) I needed some support…to help change the attitude of this employee. We didn't have anything internally to offer within the company, so we looked outside. We found something here locally, outside of the company. (Mark)
Supporting personal success	*Creating a personally-beneficial solution* In my position here, it's [difficult to move up] … So in order to offset that, other ideas were brought to the table and saying okay, why don't we get you in touch with the appropriate person so you could learn more…increase your skillset. (Susan) She…placed me in a banking center and then gave me then the opportunity to become a manager. (Jessica)
	*Providing tangible help* He goes, “Why don't we do this? Since it involves one of my managers and one of my employees… I'll have the conversation.” So he took that off of my plate. (Cindy)
	Recently I was faced with a life-changing event…so I had to go to management and say, hey my life is about to take a change…[everyone] collectively surrounded me with whatever I needed. From time away, to “don't worry about this, we got it covered” to “what do you need?” (Charles)
Trying	*Trying everything possible (e.g., connecting to a relevant authority)* He said I'm working on it, and I knew that he was working on it…he definitely knows exactly where I'm coming from (Frank)
	So I just sent her an email, and she went to HR with it, and we will know next week hopefully. (Justin)
*Insufficient action*	
Ineffective action	*Taking an action that's perceived as unhelpful* It's like a Band-Aid as opposed to like an actual fix in the system. (Cameron) I told him I will always – I will take care of you. You know you don't have to worry about that. Well [one day, he] started screaming at the top of his lungs when he's walking down the hall. (Ned)
No action	*Doing nothing when action or follow-up is expected* So they provided the opportunity to hear what I had to say, but I guess the outcome of that or the–what I hoped they had listened to didn't really take up. (James) They take it in, they understand it; the problem is that this is not implemented, and we see that. You know, they don't, they take it in, they absorb it, they are very good at, you know, this is great. I'm glad you changed your ideas, blah, blah, blah. And we get that, and unfortunately, you still don't see a change (Andy)

Table 3 displays the classifications and list of codes used to create the final conceptual model and the broader themes those codes represent. These are clustered based on a path ending, which corresponds with the closed circles in Figure 1. The stories are clustered based on three main factors: the perceived listener behavior (i.e., “was the listener initially open or closed?”), action expectation (i.e., “was action expected or was conversation enough?”) and the listener's ability to take action (i.e., “did the listener have the power to take relevant action?”)^3^.

**Table 3:** Listening path, classification, and behaviors.

**Path ending**	**Listening classification**	**Listener behavior (perceived)**	**Number of stories**	**Interviewee(s)**
1B	Shutting down	Making self-unavailable	8	Charles, Cindy, Clarissa, Curt, Danielle, Jennifer, Jessica, Ted
		Ignoring	4	Dennis, Greg, Melanie, Sarah,
		Hearing but overruling	11	Danny, Felicia, George, Jacob, Jody, Julian, Nancy, Sam, Susan, Tom, Trevor
2B	Building	Idea building	3	Cameron, Jody, Linda
		Coaching	6	Charles, Heidi (2), Jackie, Jacob, James
		Engaging on difficult topic	4	Greg, Jason, Melanie, Sam
		Showing interest	4	Curt, Jennifer, John, Trevor
4B/5A	Acting	Trying	2	Frank, Justin
5B	Acting	Using feedback	3	Andy, Nancy, Ned
		Providing tool	3	Danny, Felicia, Lauren
		Using input	11	Brian, Clarissa, Dennis, Felicia, George, Jacob, Joanna, Sarah (2), Steven, Ted
		Work-related solution creating	4	Mark, Tom, Danielle, Roger
		Personal solution creating	3	Jessica, Lauren, Susan
		Providing tangible help	4	Charles, Cindy, Julian, Omar
5C	Superficial	No action or follow-up	6	Andy, Brian, James, Joanna, Justin, Linda
		Ineffective	1	Ned
	Distracted	Ineffective	4	Cameron, Jason, John, Steven

## Findings

**Figure 2:**
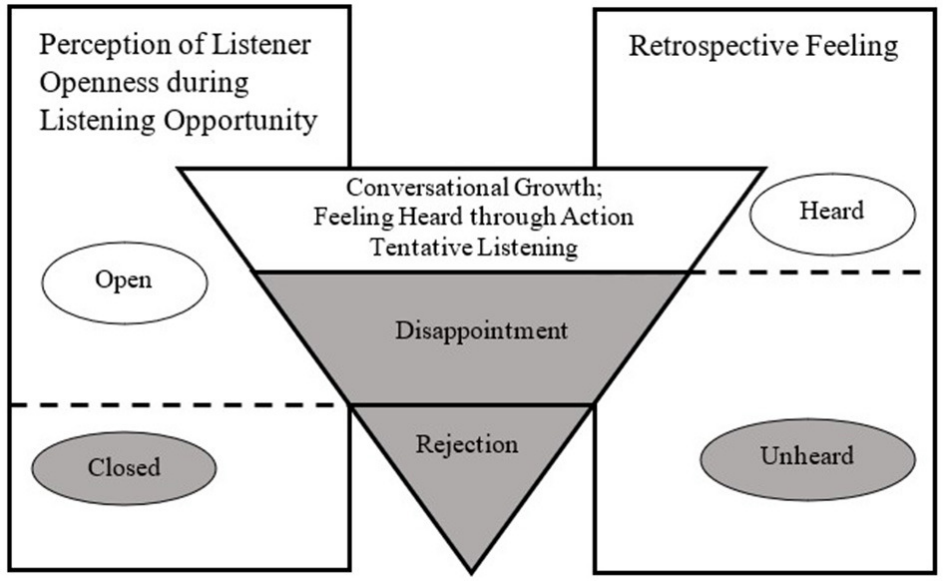
How speaker experiences can shift over the course of interaction(s) to result in feeling heard or unheard.

Stories revealed differences between two basic experiences: feeling heard and feeling unheard. Generally, speakers felt heard when listeners responded appropriately to their exchanges. In contrast, speakers felt unheard when listeners lacked response or responded in an insufficient manner. Three major themes arose that clarify how individuals subjectively experience workplace listening interactions. First, speakers reported that while listeners' attentive behavior was important, it was typically insufficient to elicit a sense of feeling heard. Instead, they reported that an attentive response that made a reasonable attempt to satisfy their situational needs (whether implicit or explicit) was the critical factor. Second, a lack of responsiveness could take passive forms, such as avoiding attending to speakers (“distracted listening”) or a failure to follow through with action (“superficial listening”), or active forms, such as immediately shutting down a request without sufficient explanation (“rejection”). Third, while responsive behavior could occur immediately, such as giving advice within conversation, many stories involved listeners who demonstrated responsiveness over an extended period. For example, Jessica described that her listener “would always keep coming back… it's not like she listened 1 day and then totally forgot about me…she would reconnect with me on a weekly basis.” All told, our data suggest that feeling heard involves responsiveness that meets a speaker's needs and expectations and that this process often spans beyond a single conversation. Sometimes an action was needed to feel heard (“feeling heard through action”), and sometimes a conversation or series of conversations was sufficient (“conversational growth”). Whether or not individuals felt heard thus depended on early listener openness and the ongoing listener responses meeting their needs and expectations. Figure 2 illustrates how listening story paths result in feeling either heard or unheard.

### Feeling Unheard

We found three different ways listeners reacted that left speakers feeling unheard, which we have labeled shutting down, superficial listening, and distracted listening. All three of these seemed to involve a violation of listening expectations; however, they differ in timing. A shutting down response (categorized below as *rejection*) was evident early on due to cues that the listener was unwilling to engage. In contrast, the other forms of non-listening were met with some optimism early on due to the listener's apparent openness; however, it became apparent later that the engagement was superficial or that a distraction had thwarted the ability to follow-up meaningfully. Thus, over time, it had become clear that an expected follow-up action was not going to be taking place. Therefore, the exchange was retrospectively classified by the interviewee as a missed opportunity for listening, leaving the speaker to feel unheard. We categorize these stories below under the label *disappointment*.

#### Rejection

Many of the stories that left interviewees feeling unheard involved *shutting down* responses in which a listener displayed a closed rather than open response, forcefully ending the listening process. These responses contained verbal and/or non-verbal signals indicating that the listener wished to end the conversation.

There were two main ways listeners shut down the conversation. First, some listeners created a barrier between speaker and listener by either making oneself unavailable for discussion or ignoring and blowing off a speaker's repeated attempts to engage. The former can be seen in the story of Charles:

I had a large commercial loan that I needed to get her to [approve]…I needed her signature because she had the lending authority. I had asked her five or six different times… I got on her calendar every time. There was something more pressing. So I felt like I was just kind of – I kept getting scooted aside and scooted aside.

Charles later described the growing impatience that he felt toward his manager at the time, the frustration that was causing his client, and the angst he felt about not better serving his client's needs. Eventually, he resorted to a more forceful approach, as did his manager:

… I put the blue memo in front of her and I'm like I need you to sign this because I need to get this thing closed…. This was in the middle of the whole process in front of my peers, and you know, because it was a pretty large request [but] I had everything in order, and she just blew, blew her top… [she said] “What do you think you're doing putting this kind of a loan request in front of me without us having to sit down on it?” She went nuts, and everybody kind of got away from her desk.

Charles walked away from her desk as well, taking the unsigned memo with him. He was eventually able to get her signature but noted that the memory of the event is still present years later when he interacts with this individual: “It's not that sharp edge where it's going to cut me, but I remember it.” The sting was connected to a violated expectation: “She should have said …I need you to sit down—let's pick a day.” Instead, the reaction he received stood out in his memory years later because of this broken expectation.

In contrast to those who reported being shut-down by a listener who had created a wall, other stories suggested that they were shut down by a would-be listener who heard overruled a speaker's perspective or request without offering the level of consideration expected by the speaker. Susan describes her experience with being overruled:

What we were asking for would have given us significantly more presence in the city along with goodwill… we jumped through so many hoops to try to do this, you know, not only for ourselves but for the organization… But when I talked to her, it was like yeah, I don't think that's anything we're going to do. That's nothing that I'm interested in. Like okay and like what can I say?

Susan described that she had been excited when she approached the manager with the idea—excited to do something for the community and the organization. Having this idea thwarted for seemingly no reason caused her to feel progressively frustrated and disengaged:

It actually negatively impacted me in regards to the amount of effort that I would give to the organization. Because they didn't care enough about me to hear my opinion and give me a valid no. I get the fact that I'm going to ask you for some ridiculous stuff and you're going to say no. I get it. But tell me why. You know I'm not asking for a pair of red Air Jordans that I could wear at work. I'm just not. It's something valid that's going to help out the business and it was just shot down… at that point I don't think I ever asked her for anything ever again. I didn't approach her for anything other than specific business relating to my office. Nothing to help further the business or increase the name reputation.

Susan describes feeling as though the response was invalid because she received no rationale for denying something she saw as a compelling opportunity for the bank. Two themes can be seen here that thread throughout other interviews as well. First, it is often most important for employees to feel listened to when trying to do something that benefits the organization somehow. Indeed, 66 of the 81 stories told (roughly 4/5) were related to an employee who was trying to do good work—ideas that would help individuals or the organizations, concerns about a work decision and the impacts it will have, suggestions for how to bring more business to the bank, etc. Yet, these good intentions seem to come at a cost. They seem to create heightened expectations around having a valid rationale for denying those contributions, as was the case above with Susan. Second, this type of “invalid” rejection leads to decreased contributions for some individuals.

#### Disappointment

Other speakers felt unheard when they were given some attention that built an expectation of a behavioral response, but were met instead with *insufficient action*, often due to the listener taking no observable action at all. The lack of response was sometimes associated with a lack of full attention early on (distracted listening) and sometimes with a perceived lack of dedication to taking relevant follow-up action (superficial listening). The latter is described by Brian:

[So we] sat down with them and said, hey, we think we have found [a previously-missed opportunity] and they just said “oh it sounds great!” but they didn't do anything about it… their actions indicated they didn't think it was that important.

Brian's experience is similar to many others: the listener appears to display attention and openness—an openness seen by speakers as a potential willingness to act by the listener. While the listener in this story acknowledged the opportunity, they did not act as Brian desired, leaving him disappointed.

Stories of feeling unheard show that the initial interaction's openness is essential for feeling heard but insufficient. Ironically, openness in an initial conversation sometimes laid the groundwork for feeling more upset later on, suggesting that speakers implicitly weighted behavioral response meeting their needs at least as heavily as in-the-moment attention.

### Feeling Heard

Although the process and content varied, two common elements appeared in those stories that left individuals feeling heard. First, speakers felt an openness from listeners during a conversation. Second, they perceived that openness translating into behavioral responses satisfying one or more of the speaker's needs, whether the need was personal, relational, or professional. Behavioral responses could occur both in those initial exchanges and much later.

We found that there were three different paths leading to the common end of feeling heard. First, many experienced outcomes as a result of the conversation(s) themselves. Stories in this category (labeled *conversational growth*) might be compared to a therapeutic listening conversation, in which conversation with an empathetic, responsive partner might leave them with new insights on self or situation. For others, their particular needs going into the conversation (or clarified through the conversation itself) implied action on the part of the listener, which they expected of the listener based on what they had shared. These stories are grouped under the label *feeling heard through action*, as the listener action was tied to whether or not they felt heard. These individuals may have felt conversational growth, but the factor distinguishing their experience was whether or not the issue was brought to its natural closure through relevant and expected action was taken by the listener. Finally, while rare within the larger collection of stories, we found two instances of individuals feeling heard while expecting an action that has not yet occurred, but which they believe is likely to occur in the future. We group these two stories under the category of *tentative listening*, and accordingly, treat these findings in a tentative way given the low prevalence within the data.

#### Conversational Growth

Stark contrasts emerged between accounts of feeling unheard and feeling heard. Among the narratives of those who felt listened to, some described that the conversation fulfilled their needs and required no further action (listeners served a *building* role). In contrast, others detailed specific follow-up actions that played a role in positively shaping their overall assessments (listeners served an *acting* role).

One way listeners facilitated conversational growth was by working with the speaker toward *expanding* ideas, capabilities, or insights. Listeners facilitated growth by helping the speaker build an idea or providing the speaker with coaching. Listeners facilitated growth also by *being with* the speaker through engaging with a difficult topic (one that might be more comfortably avoided) or showing interest when it was unexpected (which often took the form of a one-on-one meeting with a much higher-ranking individual). Collectively, we labeled these stories conversational growth because the interaction led to growth, either outwardly or internally, with the listener serving as an ally to facilitate that growth.

Coaching was a frequent theme in these stories, and this tended to come in response to discussing a career consideration of some sort. Often, the listener initiated the conversation by proactively checking in with the individual to ask about their career aspirations. Still, in some stories, the employee initiated the conversation and asked a career-related question or brought up where they saw themself going in the future. Heidi shares a story in which she talked with her manager about career options. Between her manager and the next listener she connected with, she was able to find her path and recognize the skills she needed to move into that career path:

So I started narrowing down with conversations and my boss listening to me on different things I was doing to try to explore avenues of where I wanted to go next and giving recommendations based on those conversations.

Eventually, she connected with a senior executive for a similar coaching conversation. Heidi benefitted through the mutual exploration that led to discovering her desired role. She describes the impact this had on her attitude toward work:

I mean that was definitely them listening to what I enjoy to do and what I–you know, it's something that would get me to come into work and be involved and enjoy what I'm doing, which is always going to be better work than if I'm just like punching a time card just to get paid.

In other cases, employees experienced growth from a listener who was simply willing to be there with them to have tough discussions. Greg, quoted in the introduction, reduced his communication with a manager who did not appear willing to discuss with him the difficulties he was facing in meeting the goals. Yet, Greg also describes what happens when he started working with a new manager who was more willing to have that conversation:

Honestly, he was the first person to listen to the challenges that I was having in that branch. And although he really didn't say anything from a solution standpoint, when I got into my car and I drove to my branch I made a decision right away—whatever it is that I'm doing is not working. So scrap everything that I think I know, and let's look at this for what it is—and it's non-performing.… from that moment on, everything changed.

Greg knew his performance was not what it could be, and he had wanted to talk about some of the struggles with his previous manager. But that manager's unwillingness to engage left him to be alone in the struggle. In contrast, his new manager's willingness to engage with him around the topic enabled him to find a way to turn things around.

To summarize, in these exchanges, listeners were “just listening.” Yet, these experiences left the speaker with the sense that they were walking away with something greater than what they had before the conversation. As we will see in the next sections, there were other circumstances where “just listening” was not enough, as speakers expected some form of action, based on which they deemed the experience as listening.

#### Feeling Heard Through Action

While sometimes conversation alone was enough to feel heard, in others, feeling heard was tied to action. Within these stories conversation was insufficient to elicit a sense of feeling heard because speakers needed action to accomplish a goal. Notably, interviewees commonly described this action as an integral aspect of the listening experience.

Among the simplest examples of feeling heard arose as listeners engaged in *using feedback* offered by the speaker. Stories often highlighted how interviewees offered personal feedback to a boss, colleague, or employee who internalized and applied it. Ned, an executive assistant, describes his changing impression of an executive he was serving who was new to the role. He describes finding space to give this “numbers person” some feedback about the expectations for him in his new role:

I said, “but let me tell you. [In this role] you [have to be] a prom king. You are Mr. Popular. Everybody wants a piece of you in this role. Everybody wants you to know who they are. Everybody wants you to say hello to them. They want to feel special.”

Ned's previous experience with executives enabled him to see where his new boss was lacking. This knowledge enabled him to coach his boss on becoming more approachable.

I feel like that was my purpose for him. It was to see what he was missing… I said, “You need to get out there. You need to shake hands, kiss babies”… It was really kind of cool that he took that advice and he listened to me…He's like in my own personal growth.

Like Ned's story, many of the listening stories seemed to suggest that the critical listening moments were those they felt contributed to helping the organization or the people in it. The contributions emanated from their expertise or insight due to their position or unique experiences, fulfilling a need to be validated as a provider of expertise and value. For example, Ned used the word “purpose” and referenced the mutual personal growth resulting from having his supervisor listening to his insight listened and acting upon it.

A second behavior that led speakers to feel heard occurred when listeners engaged in *facilitating good work* by providing a requested tool or using the speaker's input to make a change benefitting either the organization or individuals. Danny describes a tool that he needed to work reputably with high net-worth clients:

We have safety deposit boxes … and we used the cart to transport them from the vault into a conference room so [clients] can go through it privately. The cart was taken…. So … I was having to wheel the clients' safety deposit boxes out on a chair—so very tacky, not high net worth style.

He then recalled that he had repeatedly requested a new cart from his manager. While the manager indicated that he was working to respond, he did not fix the issue, leaving Danny frustrated at the inaction. Finally, a new manager took over and rapidly approved a new cart. Danny described the meaning that this had for him and his ability to represent the bank professionally:

I could never get this stupid cart back. He somehow, like within three days, had my cart, a brand new one … The message it sent me was “I don't care how stupid your request is, I'm your manager, I'm here to help”.

Danny's experience echoes the experience that many employees had—they were trying to do good work and positively represent the bank. Another interviewee, Brian, had an idea for better serving his customer base. He took it to progressively higher ranks until the company president ultimately approved it. Brian repeatedly noted that having the idea implemented was important to him because he was passionate about serving his customer base and, consequently, the bank:

It was important for my line of business exec, and it was important for the corporate person…because now, we're solving not just for our group, but we're solving really for the whole bank.

Finally, some speakers felt heard when listeners acted to *support personal success*. This category was used for stories in which listeners either provided tangible help or helped create a personally beneficial solution to the speaker. Often this came in the form of facilitating career goals. Jessica describes a situation in which she had regular contact with a regional manager who, through listening, helped to widen her envisioned career opportunities. Eventually, the manager used that knowledge to facilitate a desirable career move for Jessica:

At that time, having a child and working crazy hours… I'm like, alright, … I can't [keep working these hours]…she was like, what do you want to do Jessica? And she really took the time to listen and [eventually] gave me [mentoring and a different type of position]… She gave me the chance…and I grew with the company, which was a huge opportunity for me. So I don't think I would be where I'm at today if it wasn't for her.

It was not just the act of giving Jessica the job that was meaningful, but rather, the fact that this manager identified the *right* position and the *right* career trajectory for her based on insights from prior conversations. Note that both elements for feeling heard were crucial in this conversation. First, the regional manager showed sustained attention to Jessica's personal needs, which led to highly tailored insight into her optimal role and environment. When the opportunity arose, the manager acted effectively in responding to Jessica's needs.

#### Tentative Listening

Most of the important listening opportunities calling for action that the interviewees discussed were ultimately met with their desired action. However, in two cases, the interviewees classified stories as listening stories even though the ultimate, desired action had not yet been achieved. These were recent situations that were still ongoing for the interviewees. While they had not yet seen their end goal accomplished, they felt some satisfaction knowing that their listeners had done or were doing everything they could to help them reach that desired end. In one case, Frank discussed a situation in which he felt he needed more information about a promotion offered to him, but neither he nor his manager was able to get the required information:

I would say that my direct boss has definitely listened to all my concerns. I trust her a lot, and everything that I told you plus more is what I told her, and she's definitely listened to it and she gets it and I can see in her face that she wants to do more about it - but I feel like she doesn't have the pen to make that decision. So, getting back to your other question, I was like–is somebody holding up the process? I don't know the answer to that, but I know if Janet had the authority to make the decision, she would've.

In this case, Frank talks about feeling listened to and appreciating the fact that Janet understands and has done all that she can do. Still, he described perceiving that the problem was at a structural level, where there is some unknown corporate barrier preventing him from getting the information he needs to make the career decision that the bank is asking him to make. Therefore, his reaction to Janet's listening is much different from his larger reaction to the bank:

I wouldn't say it has affected my work. [But] I would say it has affected my long-term view of the organization, I mean just to put it bluntly because I wanted to make a career out of this place… I mean, I don't know how much do you want to know about me personally, but you know I've been a top producing person for [the bank] my whole career, and I feel taken advantage of, a little bit.

Frank feels listened to at the dyadic level because he trusts Janet and has a good relationship with her. But he perceives a divide between what he needs and what Janet can provide; therefore, he cannot carry over these positive, trusting feelings to the organizational level.

In another case, Justin discussed a warning that one of his employees would be leaving. At the time of the interview, he didn't yet have a final resolution. Still, he felt confident that the relevant authority figures were actively addressing his concerns, based on prior positive listening interactions.

## Discussion

Our findings capture the subjective experience of feeling heard and unheard at work. They affirm Worthington and Bodie's (2018, p. 8) observation that listening “resides in the eye of the beholder,” while shedding light on how and why objective accounts of listening interactions can differ from the subjective perspectives of speakers. Listening researchers have typically treated listening as something that is perceived behaviorally, and by extension, something that can be measured through questionnaires asking about a listener's behaviors. In contrast, when we initially coded all listener behaviors in each story, we found that the decontextualized behaviors could not reliably differentiate between experiences of feeling heard or unheard. It was only when we examined the behavior emphasized as most central to the story resolution that we were able to start seeing patterns in the data. As such, it seems that more important than the behaviors themselves to the experience of listening is how well-aligned those behaviors are to the situation and the expectations of the speaker. These results help to recast perceived listening in the workplace as a holistic experience of need responsiveness to speakers, often requiring follow-up action.

While our findings affirm and enrich listening discourse, they also help to address key unanswered questions in the workplace listening literature surrounding the role of follow-up action. To the extent that the conversation is seen to point toward relevant later action by the listener, and later action is perceived as reasonable from the speaker's perspective, later action appears to determine whether a speaker looks back upon that experience as one of feeling heard or unheard. When conversation alone was sufficient for feeling heard, we termed this experience *conversational growth*. When feeling heard was tied to action that had taken place, we labeled this experience *feeling heard through action*. Finally, when action was needed but had not yet taken place, we labeled the experience *tentative listening*.

Although follow-up action has been identified in some prior research as relevant to workplace perceptions of listening, such action has typically not been incorporated into measures of listening, most of which have been developed by relying on literatures from other disciplines (i.e., communication, psychology). Accordingly, our work sheds light on factors shaping the subjective perception of past listening interactions in the workplace context specifically, and suggests that within this context, and within the speaker's perspective, follow-up action is often important. Such insights into how employees categorize important past listening interactions are important because when an employee must decide whether to speak up about organizational ideas, offer ideas, engage creatively with work, or engage in organizational citizenship behaviors, such decisions are influenced by prior impressions of how well a listener tends to listen (e.g., Yang et al., 2021).

Our findings, therefore, enrich the understanding of listening processes in organizations. They shed light on the scope of the listening process from the speaker's perspective (often extending beyond a single conversation). They promote an understanding of how and when action plays a role in subjective listening perceptions in the workplace. Given that the subjective perception of listening has strong documented impacts on employees' workplace attitudes and behaviors (Pery et al., 2020), our work has significant implications for both listening scholarship and managerial practice.

## Conclusion

Listening is a powerful means to enhance individuals' experience and functioning at work. While scholarly insights into workplace listening have grown over recent years, expanding knowledge of the nature and impacts of listening, these insights are incomplete without asking workers how they experience feeling heard. We begin to address this imbalance and find that speakers' perspective significantly enriches the understanding of workplace listening. The widespread scholarly conception of listening as being attentive and responsive to a speaker during a single conversation is incomplete. Speakers subjectively felt heard when listeners responded attentively to their needs. When speakers were attended to carefully during a conversation but did not later follow up with the expected actions, speakers felt unheard. Therefore, our finding begs a change in the way scholars construct listening at work. Just as the old maxim states, “beauty is in the eye of the beholder,” our interviews show that Worthington and Bodie's (2018, p. 8) view that “listening is in the eye of the beholder” holds great promise in understanding listening and its importance to work.

### Contributions to Theory

This study's qualitative storytelling approach creates insight into the commonalities inherent in feeling heard. In contrast to prior accounts painting listening as something that is reflected in a set of given behavioral indicators (Bodie, 2012; Itzchakov et al., 2017; Worthington and Bodie, 2018), our findings suggest that those behaviors, on their own, are insufficient to determine whether or not a speaker will feel heard. Thus, researchers measuring perceived listening through behaviors alone are likely missing the mark to some extent if their intent is to tap into the subjective sense of feeling heard. Our work suggests that it may be prudent to focus instead on the extent to which listener responsiveness is perceived to be in alignment with speaker needs and expectations.

Our findings also clarify the various ways listening takes on meaning in the workplace contexts and in doing so, help bridge the gap between various scholarly accounts. Much of the listening scholarship portrays the power of feeling heard as tied most proximally to intrapersonal growth (which often has more distal impacts on outcomes external to the self). These perspectives tend to be rooted in Carl Rogers' seminal proclamations about listening being a profound experience facilitating personal growth and transformation (Rogers, 1959, 1980). Rogers' influences can be seen in research pointing toward impacts of listening on self-clarity (Lloyd et al., 2015), attitude clarity (Itzchakov et al., 2017, 2018), psychological safety (Castro et al., 2018), identity development (Pasupathi, 2001; Pasupathi and Billitteri, 2015), motivation (Van Quaquebeke and Felps, 2018), and well-being (Schroeder and Bergeron, 2013; Lloyd et al., 2015). Other times, researchers have focused on listening from the perspective of relational outcomes, pointing toward listening as a form of support (Jones et al., 2018; Reynolds-Kueny and Shoss, 2020; Yang et al., 2021), as a driver of intimacy and relational development (Lloyd et al., 2015; Kluger et al., 2020), or more broadly as a part of a larger social exchange process (Schroeder and Bergeron, 2013; Kluger et al., 2020). Both the intrapersonal and relational perspectives point toward listening as being meaningful for its ability to foster some sort of expansion—either intrapersonal or relational. Comparison to our findings suggests that such approaches are closely aligned with the “building” theme emerging from our research. In these building stories, it appears that the needs were epistemic. Through the conversation, the employee gained a new perspective. At times, like in Greg's conversational growth story, the listening changes the perspective of “something is wrong with my manager” to “something is wrong with me, and I'd better do something about it” (cf. Itzchakov et al., 2017; Itzchakov and Kluger, 2018).

On the other hand, some scholars have pointed toward the importance of listening for accomplishing more pragmatic outcomes external to the self, viewing listening as a critical part of coordination and communication (Worthington and Bodie, 2018). From this perspective, the accuracy of communication tends to be most important, and subjective accounts of listening matter only to the extent that they impact the individual's subsequent engagement in the communication process. Kocoglu et al. (2019) work is an example of this approach within the workplace literature, suggesting that team perceptions of listening are important because they promote faith in the ability of the team to properly coordinate actions. This approach toward listening is similar to the “acting” theme emerging from our research. Specifically, stories in which interviewees felt heard through action each involved meeting needs that depended on another individual not only taking in information, but taking tangible, cooperative action.

While these views tend to portray the importance of listening from very different perspectives, this is not necessarily reflected in the way researchers have measured listening. Specifically, action relating to a prior conversation has typically been viewed as outside the listening process by each of these scholarly camps. Moreover, a focus on what can be done to address speaker concerns has sometimes been portrayed as short-changing the needs of the speaker to autonomously generate their own insights into the issue(s) at hand (Rogers, 1951). A exception is Kocoglu et al. (2019), who argued and found support for the notion that action is an essential element of listening in the workplace team context because within teams specifically—yet notably, they did not believe action plays such a central role in dyadic workplace interactions. Indeed, the only findings we know of to suggest that action falls within the scope of dyadic workplace listening perceptions was from Lewis and Reinsch (1988) who found that immediate or delayed actions were reflected in three out of 38 themes they identified as reflecting perceptions of listening within the workplace context. Yet their analytical approach did not enable insights into how or when action matters in workplace conceptions of listening. Accordingly, researchers continued to measure listening perceptions without reference to action over the intervening three decades.

Our research helps to contextualize and shed light on these varied findings by suggesting that not all situations call for action in the speaker's eyes—in fact our “building” stories suggest that many workplace interactions are meaningful, fulfilling, and create lasting impressions based on conversational exchanges alone. Yet in other cases (the “acting” stories and “the disappointed” stories), we found that feeling heard or unheard hinged on taking action relevant to what the speaker had shared. Within these stories, action seemed to indeed “speak louder than words” (Kocoglu et al., 2019).

This focus on an appropriate match to needs has been found by researchers examining the impact of support in work relationships (Ehrhardt and Ragins, 2019), but as far as we know, this view has not been previously incorporated explicitly into the realm of listening literature. Bodie et al. (2008, p. 107–108) recognized that “the achievement (or lack thereof) of interaction goals determines the appropriateness and effectiveness of listening behavior” yet within listening research rarely are such interaction goals taken into account, and when they are, they are taken into account from the perspective of the listener (e.g., Bodie et al., 2013a; Gearhart et al., 2014). Our findings suggest this kind of nuanced view of listening experiences being tied to the speaker's needs would be fruitful for researchers to consider in conceptualizing good listening; moreover, they suggest situation-specific needs and perceived constraints play a crucial role in the overall experience of listening from the speaker's point of view.

Second, our findings point toward a variety of paths toward feeling heard or unheard, and demonstrate how listening behaviors take on meaning within each of those paths. Specifically, our findings suggest that meaningful listening interactions can help speakers meet their needs through conversation alone (e.g., providing coaching), through taking various actions (e.g., providing a requested tool), or through taking steps to facilitate the needed action (e.g., connecting with another listener who has the power to act)—yet none of these approaches are likely to produce an outcome of feeling heard if they do not match the needs and expectations of the speaker. This distinction is important from a theoretical perspective because subjective perceptions of listening have typically been measured as being tied to a given set of behaviors, regardless of the particular needs or expectations of the speaker.

Third, our work adds nuance to understanding how workplace listening may differ from listening in other contexts (e.g., therapy, friendships) by pointing toward a greater emphasis on action and extended timeframes for feeling heard regarding a single interaction topic. At work, individuals coordinate efforts to realize goals. In this context, realizing instrumental needs is at least as important, if not more important, than realizing personal needs (Ehrhardt and Ragins, 2019). Therefore, it is logical that speakers would often expect listeners to heed the information about both instrumental and personal needs they received in a conversation and respond appropriately (Kocoglu et al., 2019). Our results suggest this is often the case even at the dyadic level.

Finally, this study also shines a light on the crucial role that listening plays in supporting individuals in surfacing organizational problems and solutions. In doing so, our findings contribute to the growing literature on organizational voice and silence. Our interviewees often shared that their urgency to be heard arose from their sense that they held unique knowledge to help the organization or individuals solve problems and realize opportunities. The story of Ned, who helped the new executive grow into his position, exemplified this principle. Conversely, when interviewees felt unheard, some described that this caused them to halt future sharing (e.g., Greg). Moreover, as Susan explained, it is not necessarily the rejection itself that leaves some speakers feeling unheard, but rather how rejection is presented. Indeed, rejection accompanied by adequate rationale can serve as a form of feedback that encourages future idea sharing (Piezunka and Dahlander, 2019). Thoughtfully approached, rejection can demonstrate respect by providing closure without damaging the larger exchange of ideas.

### Practical Relevance

This study suggests several practical implications. First and foremost, these findings clarify how organizations might tailor training efforts toward listening in a way that leaves speakers feeling heard. For one, they clearly show the dangers of superficial listening, when listeners seem to attend to speakers but ultimately fail to respond to their needs. This type of exchange seemed to raise and then dash expectations. If they wish to have others feel heard, organizations must help their employees couple attention to action (where needed). Likewise, listeners may enhance the impact of their listening and signal continued openness and commitment by following up after a listening interaction.

Second, taking relevant action and following up requires perspective-taking and an anticipation of the implicit needs and expectations of the speaker. Drawing from these observations, if an employee comes to a manager with a suggestion, the manager will do well to pay attention to any hints of urgency or expected follow-up. It may also be prudent to help employees become more apparent and articulate about their needs to make listening to them easier (Itzchakov et al., 2016).

Third, research on listening styles suggests that individuals often have preferred ways of listening in interpersonal interactions (Watson et al., 1995; Bodie et al., 2013b), yet our findings suggest the possibility that listeners will be more effective in helping others feel heard to the extent that they modify their style to match the needs and expectations of the speaker. Moreover, our findings offer some preliminary insights on various ways to engage as a listener, offering several different examples of action and conversational building approaches grounded in contextualized employee experiences of feeling heard or unheard. Accordingly, such examples may prove useful in expanding the repertoire of listening approaches an individual perceives as available to them.

### Limitations and Future Studies

As with any research study, our findings are bound by certain limitations. First, our interviews were intentionally focused on retrospective accounts of subjectively feeling heard or unheard. Accordingly, we did not attempt to assess the nature of these interactions objectively, yet it would be worthwhile for future studies to triangulate between subjective accounts of feeling heard with objective analysis of listening interactions, as well as with listeners' perceptions of the same interactions. Future research may wish to capture and compare objective features of listening interactions to the subjective experience of feeling heard or unheard. Likewise, future research could also compare listeners' and speakers' subjective accounts of a given interaction.

Second, our interviews were intentionally retrospective in an attempt to gather the enduring features of interactions that left interviewees feeling heard or unheard. An intrinsic feature of this design is the substantial time between the actual incident and the report on that incident. It is possible that reducing (or even eliminating) the temporal gap between listening interaction and discussion of the experience may provide another perspective. Accordingly, future studies could be designed to shift emphasis away from the most meaningful listening exchanges toward everyday listening exchanges, in which case researchers could interview speakers shortly after a given conversational exchange. Researchers could also employ experience sampling designs, collecting data during or immediately after listening experiences.

Third, we aimed for generalizability in transferable concepts rather than aiming for generalizability in the form of replicability of our precise findings in other workplace settings (Lincoln and Guba, 1985). Accordingly, while we expect that the broad concepts identified here are likely to transfer to other contexts, we cannot guarantee that our findings would translate across the board. For example, our finding that needs and expectations are likely to shape experiences of feeling heard or unheard is likely generalizable, and likewise, we expect that in any workplace context action is likely to play a role in helping some individuals in some situations feel heard or unheard, yet the precise listening approaches identified here may vary from those identified in a different culture, and likewise, the role of action may not be as strong across all contexts. Some listening research has suggested that expectations of leader listening vary with country culture (e.g., Imhof, 2003; Es-Sabahi, 2015). Our work suggests that expectations impact the assessment of listening. Given that expectations vary across cultures, the action expectation frequently held by our interviewees may be limited to cultures similar to that of the US.

Personal characteristics could also play a role in subjective accounts of listening interactions. While beyond the scope of this research, future studies could investigate through qualitative or quantitative approaches what personal factors influence the degree to which follow up action impacts the sense of feeling heard or unheard. For example, attachment theory strongly indicates that individuals with weak attachments to parents may need to see demonstrations of need satisfaction and support to feel safe or comfortable in a given interaction (for review, see Mikulincer and Shaver, 2016). Indeed speakers, high in avoidance-attachment style may not feel heard even when their counterparts listen well (Castro et al., 2016). In contrast, those with more secure attachments may feel comparatively satisfied by attentiveness by the listener within a given conversation.

Finally, we did not directly investigate the extent to which the specific interactions discussed in these stories shaped overall perceptions of listening within these relationships. While we have reason to believe some of these interactions created lasting impressions and changed behaviors (e.g., Greg in the introductory quote), it is unclear the extent to which these impressions were shaped by these interactions alone vs. a more sustained pattern of interaction exemplified in the interactions reported by these interviewees. Further, because memories are often recalled in contextually-specific ways (Frankland et al., 2019), it could be that a story shared here in which the listener took relevant action would inform future approach behavior in situations requiring action, but that same recollection could be less relevant to shaping future approach behavior if the speaker perceives the need for conversational building only in a future interaction. Future researchers may wish to address these questions by conducting longitudinal research into dyadic listening impressions across various interaction situations, starting from the beginning of the dyadic relationship (e.g., when an employee is hired and begins working with a manager).

## Data Availability Statement

The datasets presented in this article are not readily available because the information contained could be used to identify participants. Requests to access the datasets should be directed to Tiffany D. Kriz, tiffany.kriz@macewan.ca.

## Ethics Statement

The studies involving human participants were reviewed and approved by Case Western Reserve University Institutional Review Board (CWRU IRB). The participants provided their written informed consent to participate in this study.

## Author Contributions

TK and AK contributed to the conception and design of the study. TK performed the interviews, organized and analyzed the data, and wrote the first draft of the manuscript. CL wrote a revised version of the original manuscript. All authors contributed to additional manuscript revisions and edits.

## Conflict of Interest

The authors declare that the research was conducted in the absence of any commercial or financial relationships that could be construed as a potential conflict of interest.

## Publisher's Note

All claims expressed in this article are solely those of the authors and do not necessarily represent those of their affiliated organizations, or those of the publisher, the editors and the reviewers. Any product that may be evaluated in this article, or claim that may be made by its manufacturer, is not guaranteed or endorsed by the publisher.

## Footnotes

^1^Included in this review is a discussion of whether or not action is perceived as a part of listening. Yet it is important to note that our focus on action in this manuscript was unanticipated at study conception, but rather was incorporated only after finding that action held importance in many of our interviewee accounts.

^2^Names, and in some cases, genders, have been changed in order to protect the identity of interviewees.

^3^As shown in Figure 1, a speaker could become connected with an additional listener as a part of the listening process (T1–T3), or re-engage after an unsuccessful listening experience (T4–T5). These subpaths are included in the figure to relay the process accurately, but are not discussed in the manuscript due to space constraints.
