# Supplementary material for: Feeling Heard: Experiences of Listening (or Not) at Work
Source: Front Psychol. 2021 Jul 26;12:659087. doi: 10.3389/fpsyg.2021.659087 (PMC8350774; doi:10.3389/fpsyg.2021.659087)
Supplement: Supplementary file 1 [file Data_Sheet_1.PDF]

Appendix

Interview Protocol

Another purpose of this study is to find out more about how listening actually happens in organizations, and to understand when being listened to by others is most impactful. We know that it's important for employees to feel that someone is listening to them at work, but there is little research to help us understand when it matters most for people to feel that someone in their workplace is listening to them. So I'd like to hear from you about those moments when someone's listening (or a lack thereof) made a lasting impression on you.

Tell me about a time when someone at work had an important opportunity to listen to you, and he/she took that opportunity.

Tell me about a time when someone at work had an important opportunity to listen to you, but he/she failed to take full advantage of that opportunity.

- Potential probes:
    - What happened?
    - Who was involved?
    - Where were you?
    - What made this stand out as important in your mind?
    - How did you react?
    - What was the consequence?
